# Effects of Tai Chi and Baduanjin on muscle mass, muscle function, and activities of daily living in individuals with sarcopenia: a systematic review and meta-analysis

**DOI:** 10.3389/fpubh.2025.1636857

**Published:** 2025-09-03

**Authors:** Sen Yang, Shuwei Chen, Yong Zhang, Zhixiong Zhou, Donghai Li, Ping Zeng

**Affiliations:** ^1^Institute for Sport Performance and Health Promotion, Capital University of Physical Education and Sports, Beijing, China; ^2^State Key Laboratory of Complex, Severe, and Rare Diseases, Institute of Basic Medical Sciences, Chinese Academy of Medical Sciences and School of Basic Medicine, Peking Union Medical College, Beijing, China; ^3^Institute of Artificial Intelligence in Sports, Capital University of Physical Education and Sports, Beijing, China; ^4^Department of Burn, Plastic and Reconstructive Surgery, and Wound Repair, Beijing Fengtai You'anmen Hospital, Beijing, China; ^5^Health and Medical Department, Peking Union Medical College Hospital, Chinese Academy of Medical Science, Beijing, China

**Keywords:** Tai Chi, Baduanjin, sarcopenia, systematic review, meta-analysis

## Abstract

**Objective:**

This study aims to systematically evaluate the effects of Tai Chi and Baduanjin on muscle mass, muscle function, and activities of daily living (ADL) in patients with sarcopenia.

**Methods:**

We conducted a comprehensive literature search across both English and Chinese databases, including the Cochrane Library, PubMed, Embase, CNKI, Wanfang, and VIP, for randomized controlled trials (RCTs) examining the effects of Tai Chi and Baduanjin on patients with sarcopenia. The search covered all studies from the inception of the databases through March 28, 2025. We performed a meta-analysis using RevMan 5.3 and Stata 15.1 software.

**Results:**

A total of 11 RCTs involving 738 participants were included in the meta-analysis. The overall risk of bias was assessed as low, with the methodological quality of the studies ranging from moderate to low-moderate. Compared to controls, both Tai Chi and Baduanjin demonstrated positive improvements in grip strength (SMD = 0.97, 95% CI: 0.42 to 1.52, Z = 3.45, *p* = 0.001), gait speed (WMD = 0.10, 95% CI: 0.02 to 0.19, Z = 2.47, *p* = 0.013), and muscle strength (WMD = 1.75, 95% CI: 0.59 to 2.91, Z = 2.95, *p* = 0.003). However, changes in skeletal muscle mass index (WMD = 0.55, 95% CI: −0.54 to 1.65, *Z* = 0.99, *p* = 0.323) and ADL (WMD = 11.04, 95% CI: −2.08 to 24.16, *Z* = 1.65, *p* = 0.099) were not accompanied by significant changes. The funnel plots appeared largely symmetrical, indicating minimal concern for publication bias across the primary outcomes. Furthermore, Egger’s tests for grip strength (*t* = 0.41, *p* = 0.695), gait speed (*t* = 1.37, *p* = 0.265), and skeletal muscle mass index (*t* = 3.16, *p* = 0.087) showed no significant publication bias.

**Conclusion:**

Both Tai Chi and Baduanjin significantly improve muscle strength and function in patients with sarcopenia. However, the improvements observed in ADL did not reach statistical significance.

**Systematic review registration:**

https://www.crd.york.ac.uk/PROSPERO/view/CRD420251032762, Identifier CRD420251032762.

## 1 Introduction

Sarcopenia is a common age-related disorder characterized by the gradual loss of muscle mass, strength, and physical function, particularly among older adults and those with limited physical activity. The development of sarcopenia involves multiple contributing factors, including disuse-related muscle atrophy, hormonal shifts, poor nutrition, and chronic inflammation (1). Sarcopenia is estimated to affect nearly one in three older adults in community care settings, with prevalence rising up to 50% in those over the age of 80 (2).

Currently, there are no FDA-approved medications specifically for treating sarcopenia. Nonetheless, several therapeutic agents have been investigated, including growth hormone, anabolic and androgenic steroids, selective androgen receptor modulators (SARMs), protein synthesis enhancers, appetite stimulants, myostatin inhibitors, activin type II receptor antagonists, *β*-adrenergic blockers, angiotensin-converting enzyme inhibitors, and troponin activators. Despite this, herbal supplements - such as curcumin, alkaloids, catechins, proanthocyanidins, gingerols, and sage - have demonstrated limited efficacy in improving skeletal muscle function (3).

Given the lack of effective drug therapies, exercise remains a key component of sarcopenia management, with strong evidence supporting its role in enhancing strength, mass, and mobility (4, 5). Among traditional Chinese exercises, Tai Chi and Baduanjin have shown potential in managing sarcopenia. Tai Chi emphasizes slow, deliberate movements paired with deep breathing, helping to improve balance, coordination, and muscle control (6). Baduanjin is a traditional qigong practice made up of eight repetitive movements designed to enhance qi, improve musculoskeletal health, and support physical function (7). While Tai Chi and Baduanjin show promise, existing research on their impact in sarcopenic populations is limited and often inconsistent. Existing studies report inconsistent outcomes regarding improvements in muscle mass, muscle function, and activities of daily living (ADL). To address these gaps, we conducted a meta-analysis to evaluate the effects of Tai Chi and Baduanjin on muscle mass, muscle function, and ADL in patients with sarcopenia, aiming to inform clinical practice through evidence-based recommendations.

## 2 Materials and methods

### 2.1 Search strategy

We systematically searched English and Chinese databases - Cochrane Library, PubMed, Embase, China National Knowledge Infrastructure (CNKI), Wanfang Data, and VIP - to identify relevant studies. The search spanned from the inception of each database through March 28, 2023. To maximize retrieval sensitivity, we used a combination of controlled vocabulary (subject terms) and free-text terms. Additionally, we manually screened the reference lists of relevant articles to identify any additional eligible studies. The search terms included: sarcopenia, Baduanjin, qigong, Taiji, Tai Chi, and Tai Chi Chuan. This study is registered with PROSPERO (CRD420251032762).

### 2.2 Inclusion and exclusion criteria

Inclusion criteria:

1. Study type: RCTs assessing the effects of Tai Chi or Baduanjin on muscle mass, muscle function, and ADL in individuals with sarcopenia.
2. Participants: Individuals clinically diagnosed with sarcopenia.
3. Intervention: Intervention groups received Tai Chi or Baduanjin; control groups received routine physical activity, health education, or standard care.
4. Outcomes: Primary outcome measures included muscle mass, muscle function, and ADL.

Exclusion criteria:

1. Non-RCT studies or those not meeting diagnostic or intervention criteria for sarcopenia.
2. Studies with vague outcome definitions, misclassified variables, or flawed statistical analyses.
3. Studies lacking extractable or complete outcome data.

### 2.3 Literature screening and data extraction

Two reviewers independently screened all records retrieved from the databases. We first evaluated titles and abstracts against the predefined inclusion and exclusion criteria. For studies deemed potentially eligible, we conducted a full-text review. Final inclusion was determined by consensus between the two reviewers; disagreements were resolved through consultation with a third reviewer. From each included study, we extracted detailed information on study characteristics, participant sources, sample size, intervention protocols, and other relevant variables.

### 2.4 Quality assessment

We assessed the risk of bias in the included studies according to the criteria outlined in the *Cochrane Handbook for Systematic Reviews of Interventions* (version 5.1.0). The assessment covered seven domains: random sequence generation, allocation concealment, blinding, attrition reporting, completeness of outcome data, selective reporting, and other potential biases. Based on these criteria, we assigned studies to one of three quality grades: Grade A (low risk of bias, meeting all seven criteria), Grade B (moderate risk, meeting some criteria), and Grade C (high risk, meeting few or none of the criteria).

### 2.5 Statistical analysis

We conducted statistical analyses using Stata 15.1 and RevMan 5.3. To evaluate heterogeneity, we used the *I^2^* statistic and Cochran’s *Q* test. When *I^2^* > 50% and *p* < 0.1, we considered heterogeneity substantial and applied a random-effects model for the meta-analysis. If *p* > 0.1 and *I*^2^ ≤ 50%, we assumed homogeneity and used a fixed-effects model. We also performed sensitivity analyses (*α* = 0.05) and subgroup analyses to explore potential sources of heterogeneity. To assess publication bias, we used funnel plots and Egger’s test.

## 3 Results

### 3.1 Literature search results and study characteristics

**Figure 1:**
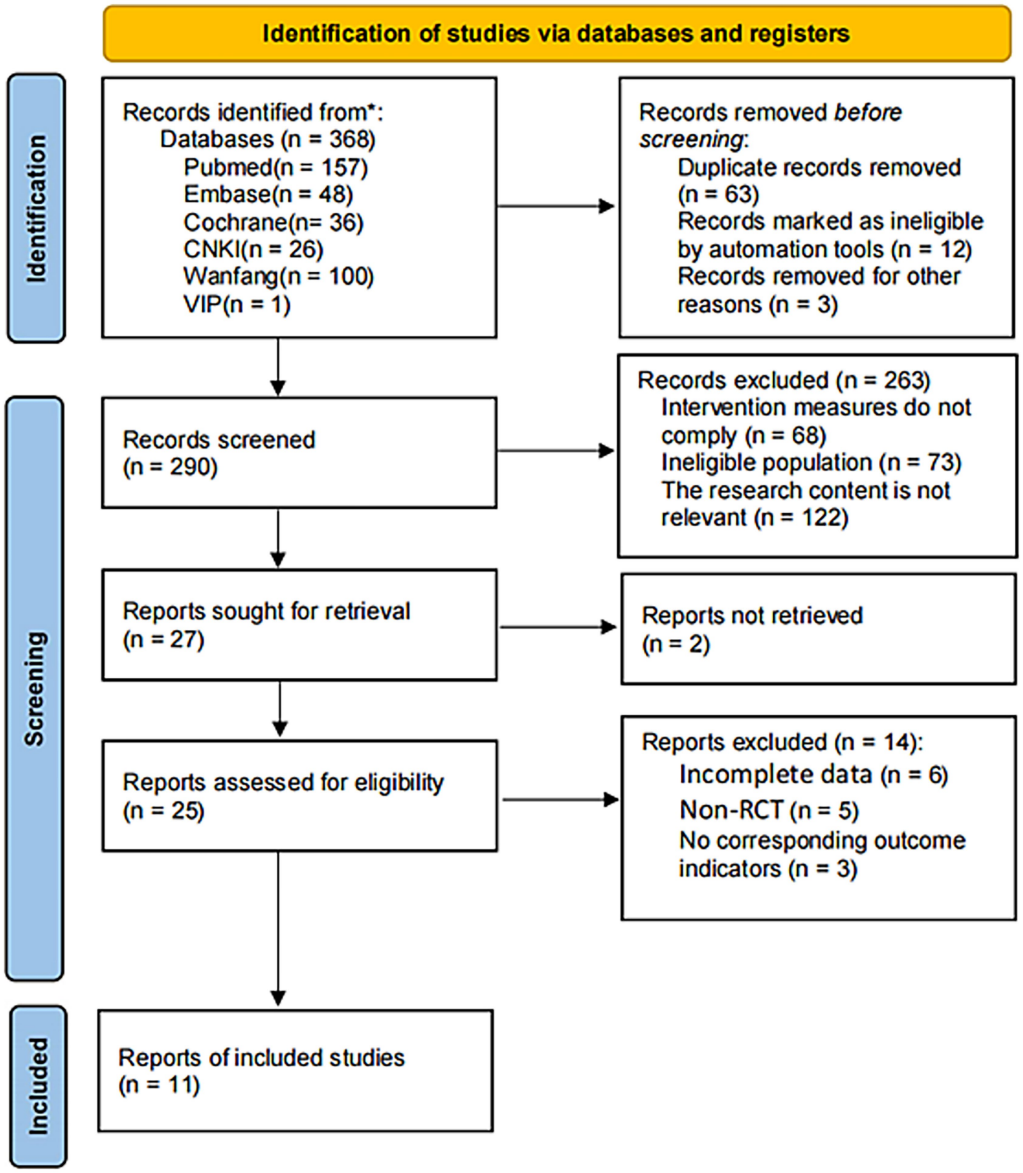
PRISMA flow diagram for the selection of studies.

We initially identified 368 relevant articles. After a stepwise screening process, we included 11 studies in the final analysis. Figure 1 illustrates the literature selection process. Table 1 summarizes the basic characteristics of the included studies.

**Table 1:** Baseline characteristics of participants included in the study.

Included study	Sample size (Intervention/Control)	Intervention protocol	Exercise frequency (per week)	Duration (weeks)	Age (Intervention/Control)	Outcome measures
Wang et al. (42)	33/35	Baduanjin Exercise	3	12	62.2 ± 10.0/62.1 ± 9.8	①②③
Meng et al. (43)	26/25	AI-Guided Remote Baduanjin	3	12	71.15 ± 7.95/70.77 ± 8.57	①②
Carcelén-Fraile et al. (44)	63/62	Baduanjin Exercise	2	12	69.7 ± 6.15/69.75 ± 6.76	④
Peng et al. (45)	63/63	Baduanjin Combined with Blood Flow Restriction Training	5	12	70.65 ± 5.78	①③④⑤
Huang et al., 2024 (46)	40/40	Baduanjin Combined with Evidence-Based Precision Nursing	2	12	72.14 ± 8.34	③
Xu et al. (47)	30/30	Baduanjin Combined with Resistance Training	3	12	70.63 ± 0.85/70.73 ± 0.76	①②⑤
Chen et al. (48)	30/30	Baduanjin Combined with Buzhong Yiqi Decoction	7	13	72.12 ± 3.33/71.94 ± 3.40	①②
Wu et al. (49)	58/57	Baduanjin Exercise	2	12	57.12 ± 11.56/56.98 ± 10.67	①②
Huang et al. (50)	27/29	Tai Chi	3	12	69.70 ± 5.05/72.14 ± 4.79	-
Guo et al. (51)	33/30	Tai Chi Combined with Exercise Training	3	24	66.94 ± 4.42/65.42 ± 3.97	①③
Zhu et al. (52)	30/30	Tai Chi	5	72	64 ± 3/64 ± 4	-

① Grip strength (kg); ② Gait speed (m/s); ③ Skeletal muscle mass index (kg/m^2^); ④ Muscle strength; ⑤ Daily living activities; “–” indicates not reported.

### 3.2 Quality assessment

All included studies were randomized trials, though only 7 clearly described their randomization methods, and 4 reported using allocation concealment. While only 3 studies mentioned participant blinding, blinding of outcome assessors is generally more practical in such interventions; 2 studies reported employing blinded outcome assessment. Based on our quality assessment, 3 studies met all methodological criteria and were rated as Grade A, indicating low risk of bias. The remaining 8 studies were rated as Grade B, indicating moderate risk of bias. All studies confirmed baseline comparability between intervention and control groups. Overall, the methodological quality of the included studies was moderate to high. Table 2 provides a detailed summary of the quality assessment.

**Table 2:** Assessment of methodological quality for the included studies.

Included study	Randomization method	Allocation concealment	Blinding		Completeness of outcome data	Selective reporting bias	Other bias	Quality rating
			Participants	Assessors				
Wang et al. (42)	HR	HR	UR	UR	LR	UR	LR	B
Meng et al. (43)	LR	LR	UR	UR	UR	HR	LR	B
Carcelén-Fraile et al. (44)	LR	LR	LR	UR	LR	LR	LR	A
Peng et al. (45)	LR	LR	UR	UR	UR	UR	UR	B
Huang et al. (46)	LR	UR	HR	HR	UR	UR	LR	B
Xu et al., 2022 (47)	HR	HR	HR	HR	LR	UR	LR	B
Chen et al., 2022 (48)	LR	UR	HR	HR	UR	UR	UR	B
Wu et al. (49)	HR	HR	UR	UR	UR	UR	UR	B
Huang et al., 2023 (50)	LR	LR	LR	LR	LR	LR	LR	A
Guo et al. (51)	LR	UR	LR	LR	LR	LR	UR	A
Zhu et al. (52)	HR	UR	UR	HR	UR	UR	UR	B

High Risk, HR; Low Risk, LR; Unclear Risk, UR.

### 3.3 Effects of Tai Chi and Baduanjin on muscle function in patients with sarcopenia

#### 3.3.1 Effects on grip strength

**Figure 2:**
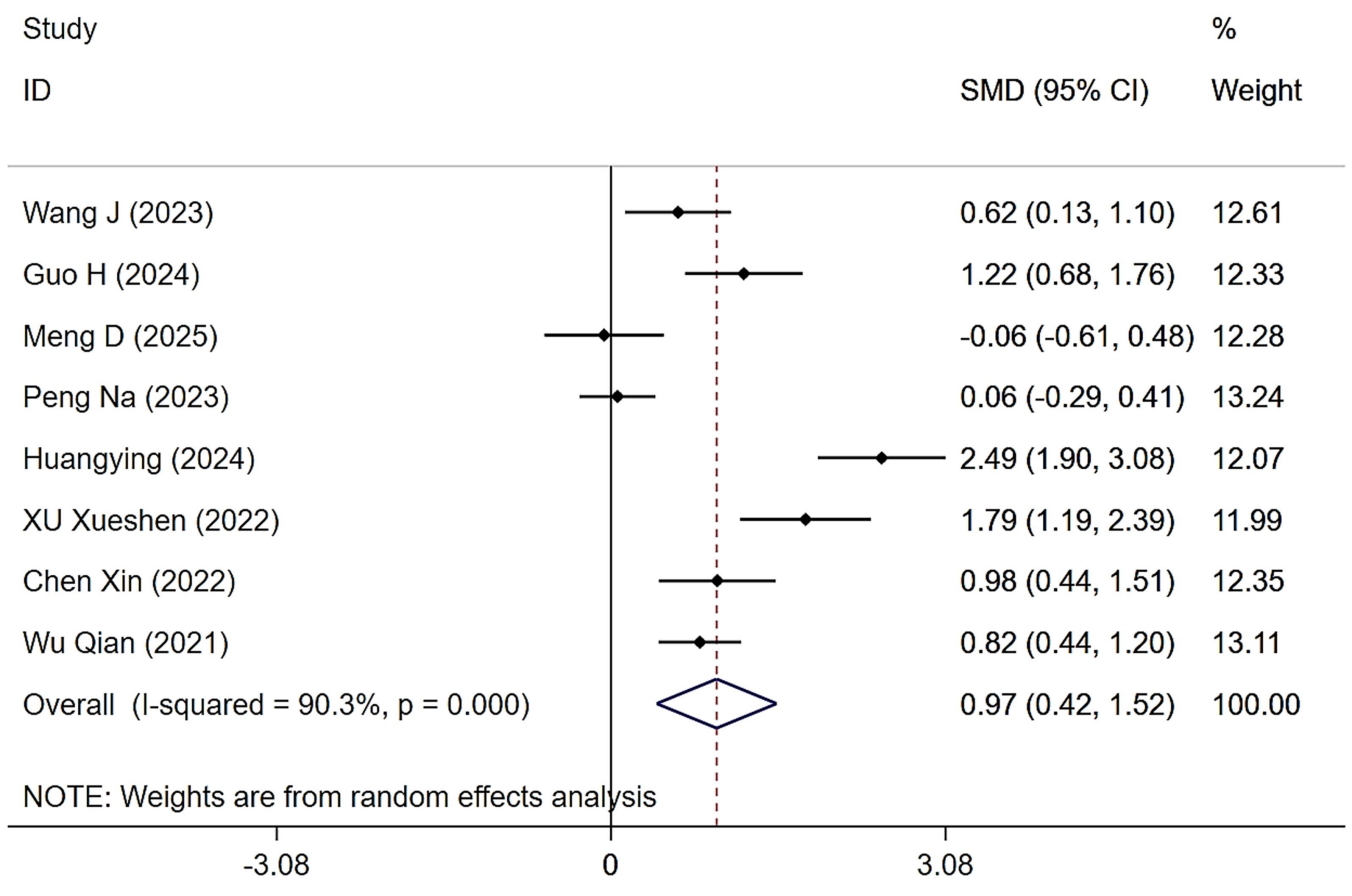
Forest plot of the effects of Tai Chi and Baduanjin on grip strengths in patients with sarcopenia.

Eight studies assessed the impact of Tai Chi and Baduanjin on grip strength in individuals with sarcopenia. Heterogeneity analysis revealed considerable variability among studies (*Q* = 72.39, (d.f. = 7), *p* < 0.0001, *I^2^* = 90.3%), necessitating the use of a random-effects model for meta-analysis. As shown in Figure 2, participants in the Tai Chi and Baduanjin group showed significantly greater improvements in grip strength compared to those in the control group (SMD = 0.97, 95% CI: 0.42 to 1.52, *Z* = 3.45, *p* = 0.001). Given the high degree of heterogeneity, we conducted a sensitivity analysis. Excluding the study by Huang et al. *I*^2^ was reduced from 90.3 to 84%, suggesting a modest decrease in heterogeneity, although it remained substantial. To further explore potential sources of variation, we performed subgroup analyses based on intervention modality, exercise frequency, and intervention duration.

##### 3.3.1.1 Effects of Tai Chi and Baduanjin on grip strength based on different intervention modalities

**Figure 3:**
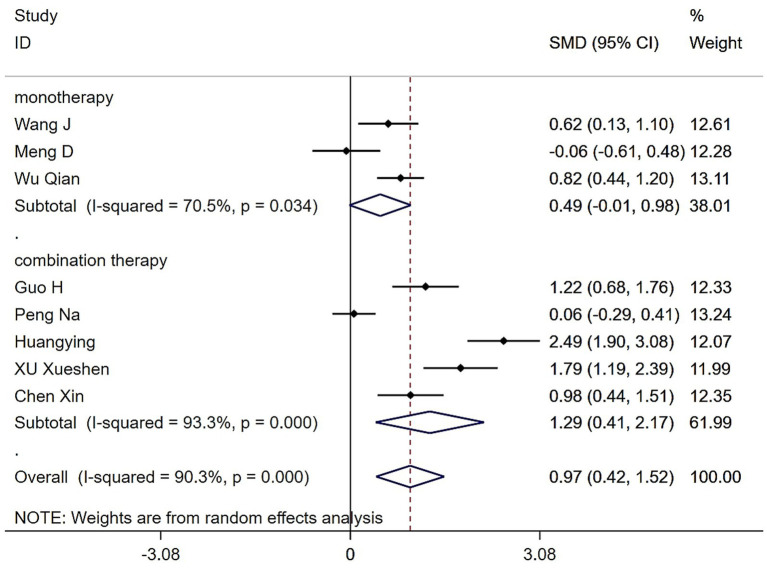
Forest plot of the effects of various Tai Chi and Baduanjin intervention modalities on grip strengths in patients with sarcopenia.

Subgroup analysis of different intervention modalities revealed that both single and combined forms of Tai Chi and Baduanjin significantly enhanced grip strength in patients with sarcopenia compared to control groups. The overall pooled effect size was statistically significant (SMD = 0.97, 95% CI: 0.42 to 1.52, Z = 3.45, *p* = 0.001). Three studies assessed Tai Chi or Baduanjin as standalone interventions, with moderate heterogeneity observed (*Q* = 6.77, (d.f. = 2), *p* = 0.034, *I^2^* = 70.5%), warranting the use of a random-effects model. The pooled effect size for this subgroup was not statistically significant (SMD = 0.49, 95% CI: −0.01 to 0.98, *Z* = 1.91, *p* = 0.056). In contrast, five studies incorporated Tai Chi or Baduanjin with other interventions, exhibiting substantial heterogeneity (*Q* = 59.87, (d.f. = 4), *p* < 0.0001, *I^2^* = 93.3%), and a random-effects model was again applied. The combined intervention group demonstrated a statistically significant improvement in grip strength (SMD = 1.29, 95% CI: 0.41 to 2.17, *Z* = 2.86, *p* = 0.004) (Figure 3).

##### 3.3.1.2 Effects of Tai Chi and Baduanjin on grip strength at different exercise frequencies

**Figure 4:**
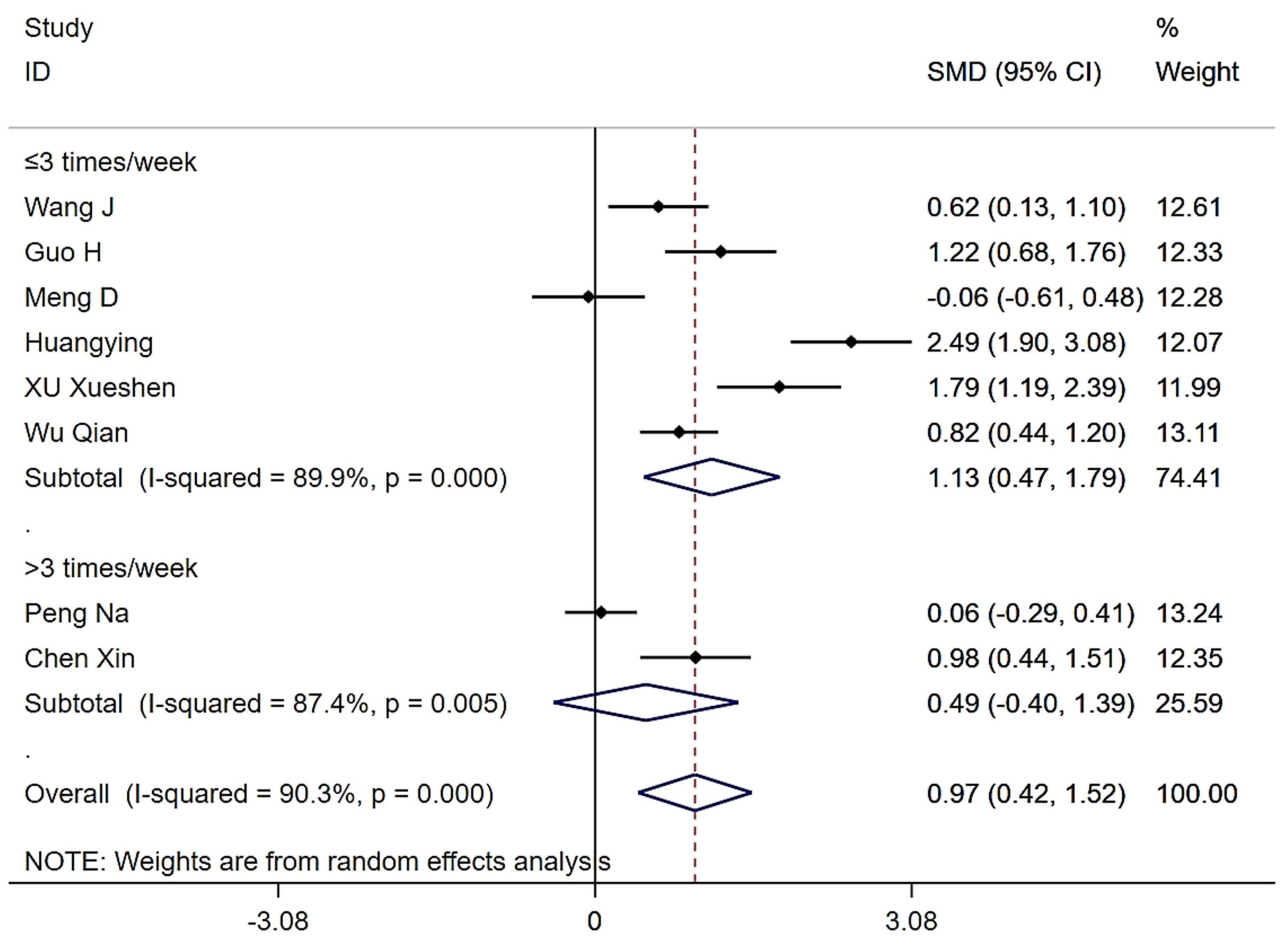
Forest plot of the effects of various exercise frequencies of Tai Chi and Baduanjin on grip strengths in patients with sarcopenia.

Subgroup analysis based on exercise frequency revealed that both Tai Chi and Baduanjin interventions significantly enhanced grip strength in patients with sarcopenia compared to control groups. Among six studies with exercise ≤ 3 times/week, between-group heterogeneity was high (*Q* = 49.63, (d.f. = 5), *p* < 0.0001, *I^2^* = 89.9%). Using a random-effects model, the difference remained statistically significant (SMD = 1.13, 95% CI: 0.47 to 1.79, *Z* = 3.37, *p* = 0.001). In contrast, two studies with exercise > 3 times/week also showed substantial heterogeneity (*Q* = 7.92, (d.f. = 1), *p* = 0.005, *I^2^* = 87.4%). Under a random-effects model, the between-group difference was not statistically significant (SMD = 0.49, 95% CI: −0.40 to 1.39, *Z* = 1.08, *p* = 0.281) (Figure 4).

##### 3.3.1.3 Effects of Tai Chi and Baduanjin on grip strength based on exercise duration

**Figure 5:**
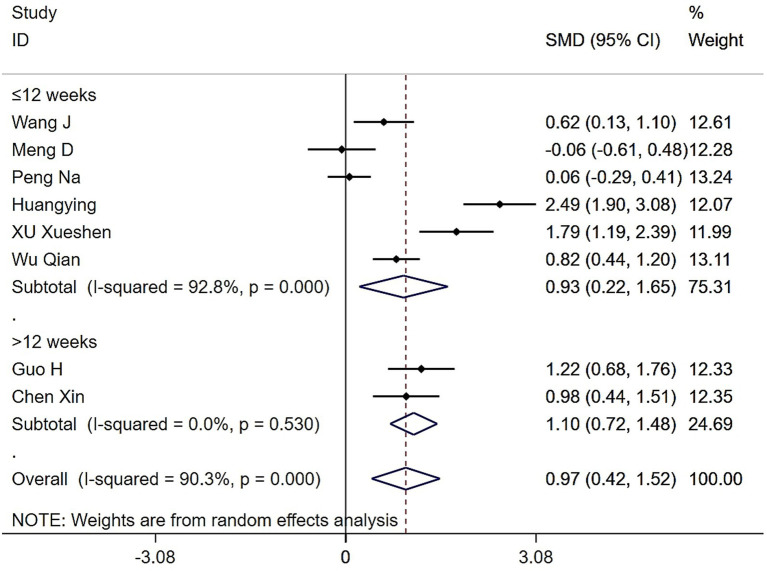
Forest plot of the effects of various intervention durations of Tai Chi and Baduanjin on grip strengths in patients with sarcopenia.

Subgroup analysis stratified by exercise duration also demonstrated that Tai Chi and Baduanjin significantly improved grip strength in patients with sarcopenia compared to controls. The overall pooled effect remained statistically significant (SMD = 0.97, 95% CI: 0.42 to 1.52, *Z* = 3.45, *p* = 0.001). Six studies employed interventions lasting ≤12 weeks, which showed substantial heterogeneity (*Q* = 69.1, (d.f. = 5), *p* < 0.0001, *I*^2^ = 92.8%). Using a random-effects model, we found a significant improvement in grip strength in this subgroup (SMD = 0.93, 95% CI: 0.22 to 1.65, *Z* = 2.56, *p* = 0.011). Two studies implemented interventions longer than 12 weeks, which exhibited low heterogeneity (*Q* = 0.4, (d.f. = 1), *p* = 0.530, *I*^2^ = 0.0%). The pooled effect size for this subgroup was also statistically significant (SMD = 1.10, 95% CI: 0.72 to 1.48, *Z* = 5.66, *p* < 0.0001). These findings suggest that variation in intervention duration may contribute to heterogeneity in grip strength outcomes (Figure 5).

#### 3.3.2 Effects of Tai Chi and Baduanjin on gait speed in patients with sarcopenia

**Figure 6:**
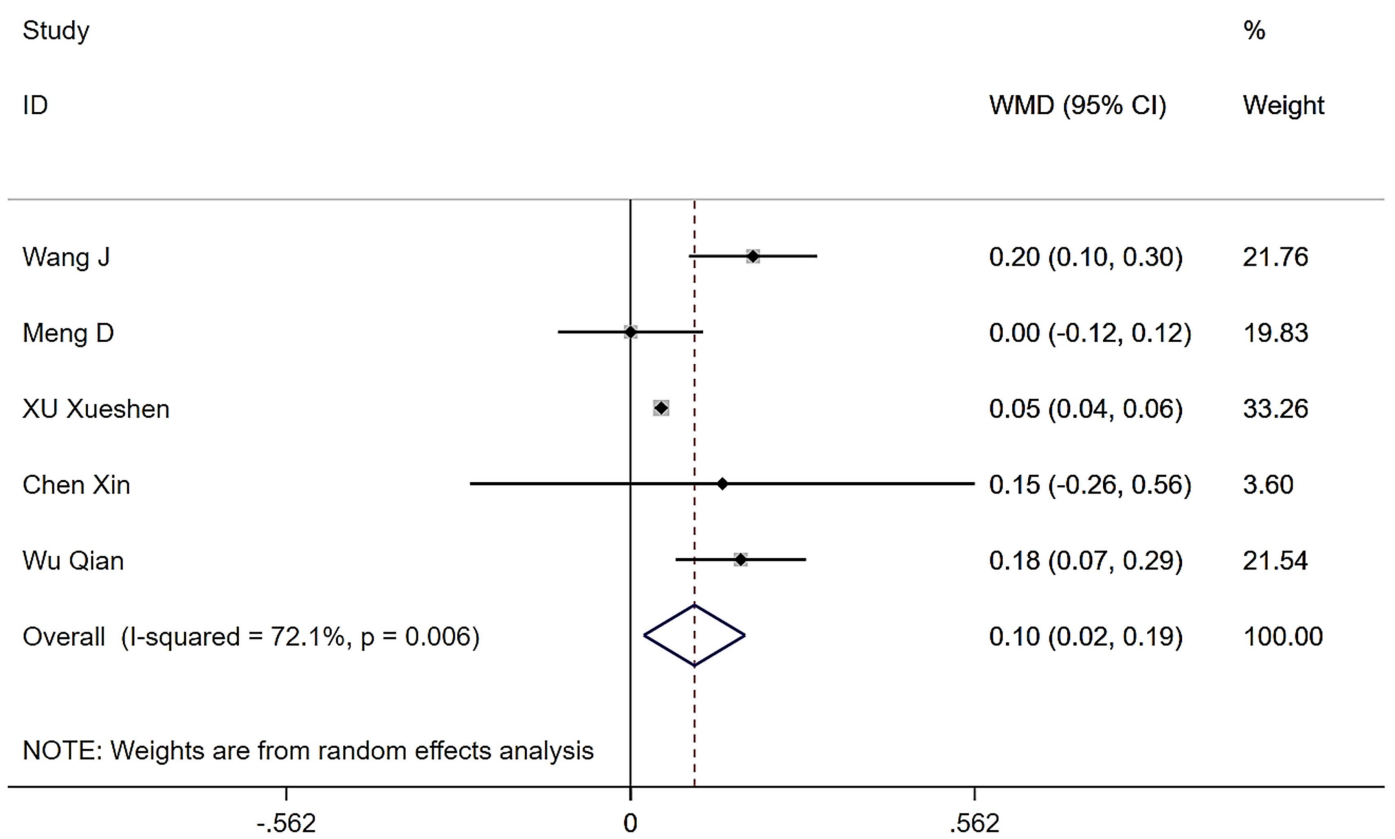
Forest plot of the effects of Tai Chi and Baduanjin on gait speeds in patients with sarcopenia.

Eight studies examined changes in gait speed following Tai Chi or Baduanjin. Results showed moderate heterogeneity across studies (*Q* = 14.34, (d.f. = 4), *p* = 0.006, *I*^2^ = 72.1%), hence we used a random-effects model for the meta-analysis. The results demonstrated that the Tai Chi and Baduanjin group significantly improved gait speed compared to the control group (WMD = 0.10, 95% CI: 0.02 to 0.19, *Z* = 2.47, *p* = 0.013) (Figure 6). To investigate the source of heterogeneity, we conducted a sensitivity analysis. Excluding the study by Wang et al. reduced *I^2^* from 72.1 to 55%, indicating that this study contributed substantially to the overall heterogeneity.

### 3.4 Effects of Tai Chi and Baduanjin on muscle mass in patients with sarcopenia

#### 3.4.1 Effects on skeletal muscle mass index (SMI)

**Figure 7:**
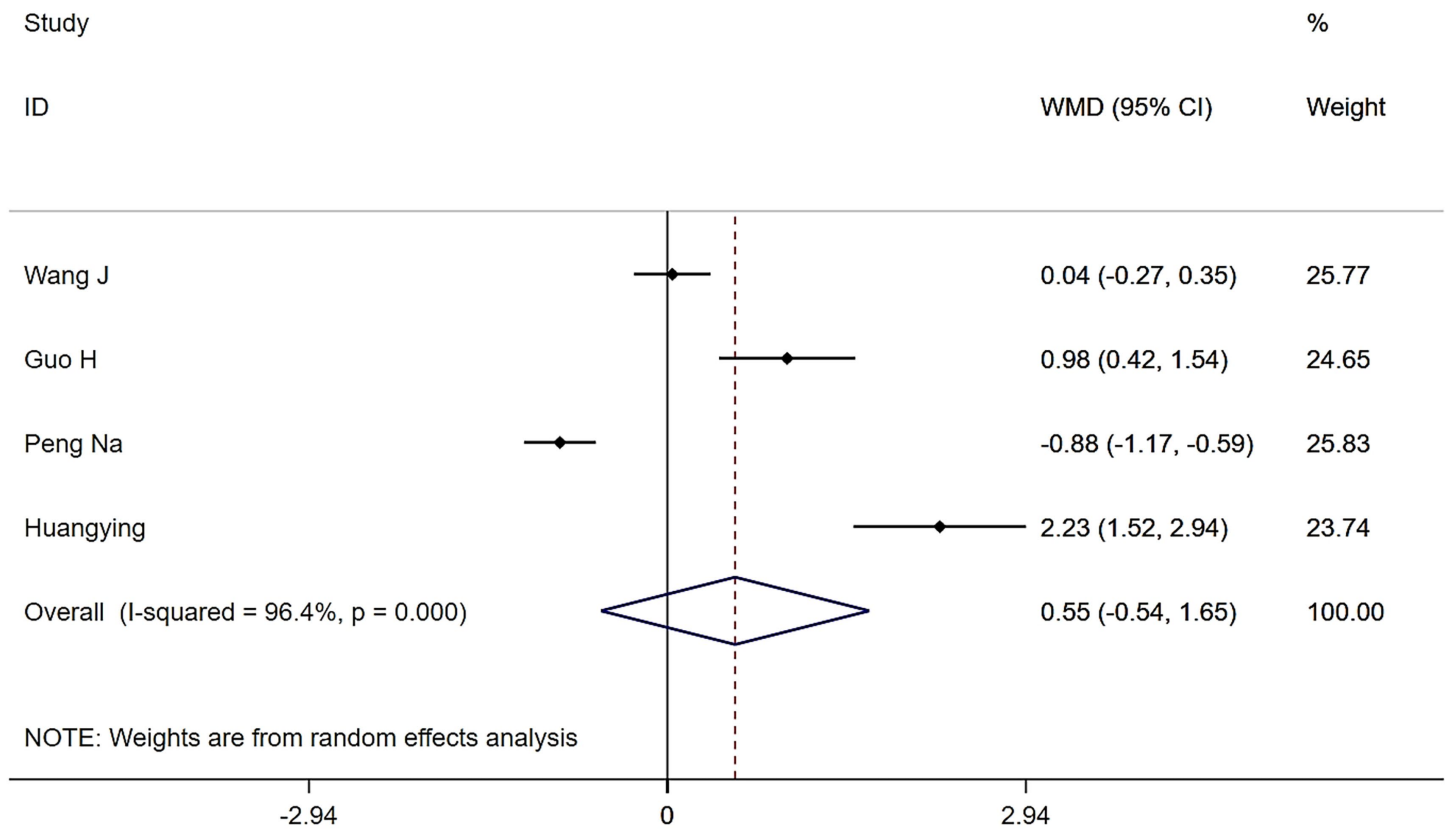
Forest plot of the effects of Tai Chi and Baduanjin on skeletal muscle mass indices in patients with sarcopenia.

Four studies examined the effects of Tai Chi and Baduanjin on the SMI in patients with sarcopenia. High heterogeneity was observed (*Q* = 84, (d.f. = 3), *p* < 0.0001, *I^2^* = 96.4%), prompting the use of a random-effects model. While participants in the Tai Chi and Baduanjin groups experienced modest gains in SMI, these improvements did not reach statistical significance compared to controls (WMD = 0.55, 95% CI: −0.54 to 1.65, *Z* = 0.99, *p* = 0.323) (Figure 7). Sensitivity analyses confirmed that heterogeneity remained high, even when studies were removed one by one.

#### 3.4.2 Effects of Tai Chi and Baduanjin on muscle strength in patients with sarcopenia

**Figure 8:**
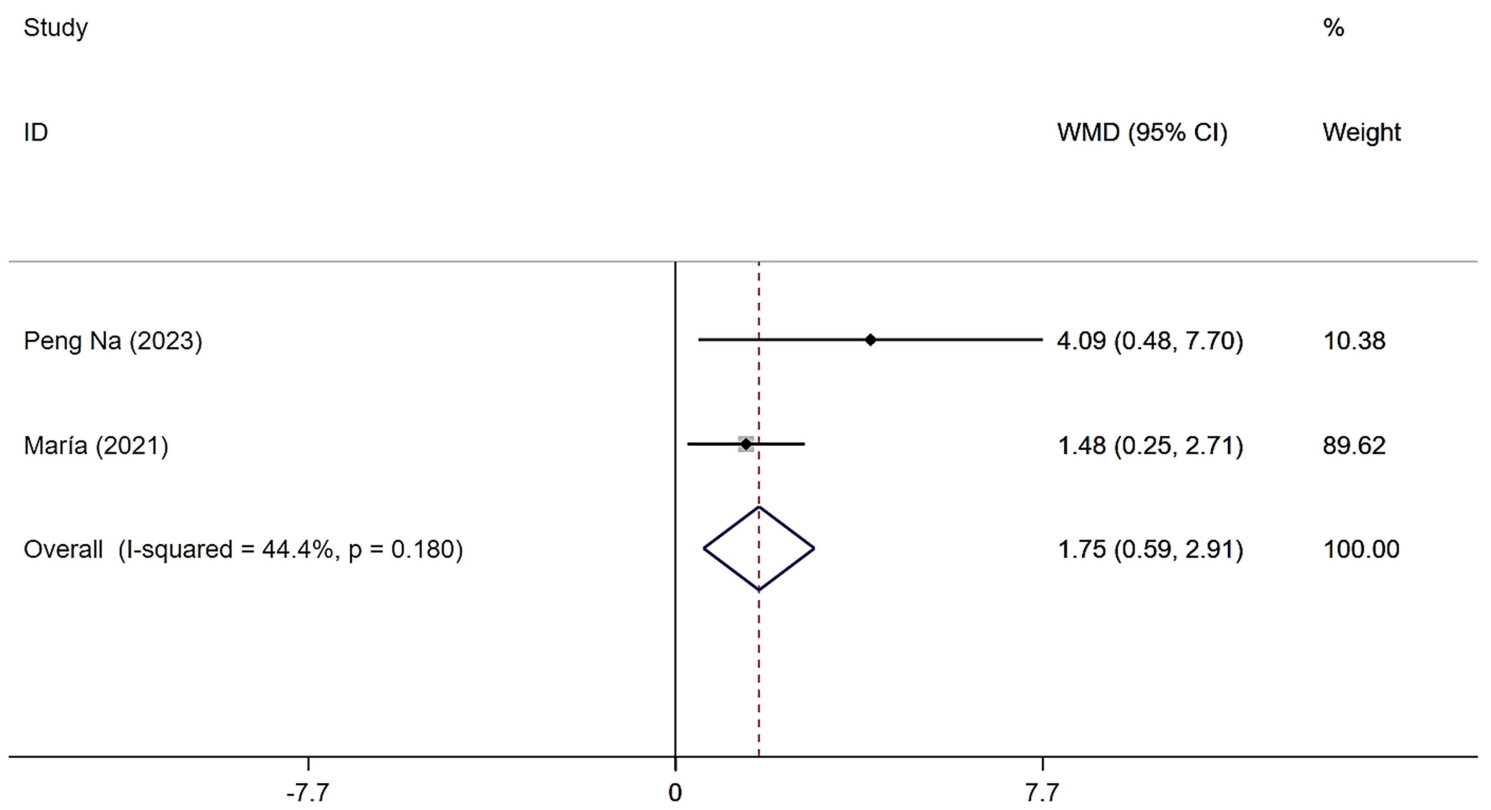
Forest plot of the effects of Tai Chi and Baduanjin on muscle strength in patients with sarcopenia.

Only two studies assessed the effects of Tai Chi and Baduanjin on muscle strength in patients with sarcopenia. Heterogeneity testing revealed low to moderate heterogeneity (*Q* = 1.8, (d.f. = 1), *p* = 0.180, *I*^2^ = 44.4%), and we applied a fixed-effects model for the meta-analysis. The results showed that the Tai Chi and Baduanjin group significantly improved muscle strength compared to the control group, with a statistically significant effect (WMD = 1.75, 95% CI: 0.59 to 2.91, *Z* = 2.95, *p* = 0.003) (Figure 8).

### 3.5 Effects of Tai Chi and Baduanjin on daily living activities in patients with sarcopenia

**Figure 9:**
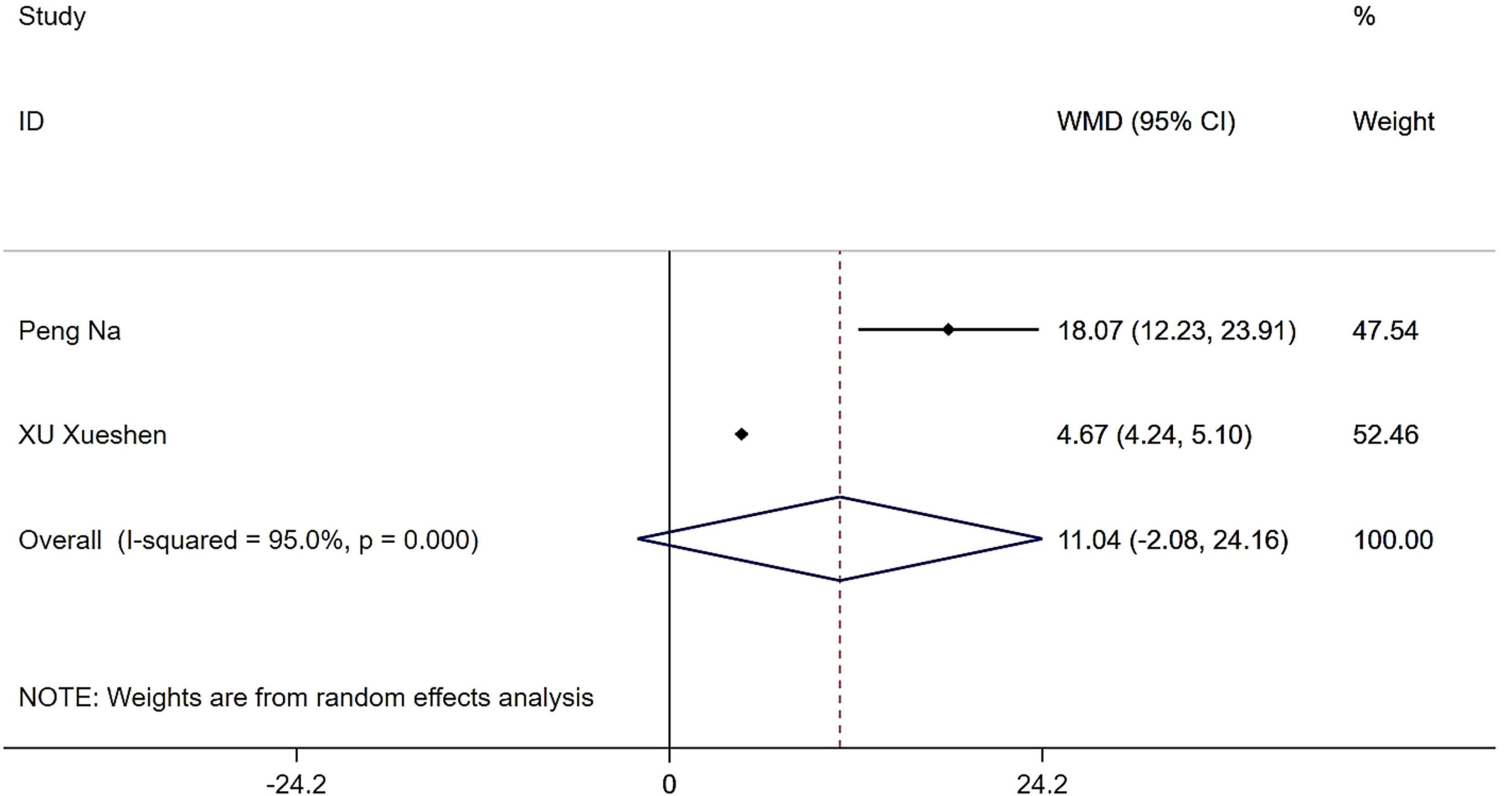
Forest plot of the effects of Tai Chi and Baduanjin on daily living activities in patients with sarcopenia.

Two studies evaluated the effects of Tai Chi and Baduanjin on ADL in patients with sarcopenia. Heterogeneity testing revealed substantial heterogeneity (*Q* = 20.11, (d.f. = 1), *p* < 0.0001, *I*^2^ = 95%), prompting the use of a random-effects model. The results showed that although the Tai Chi and Baduanjin group demonstrated greater improvement in ADL than the control group, the difference was not statistically significant (WMD = 11.04, 95% CI: −2.08 to 24.16, *Z* = 1.65, *p* = 0.099) (Figure 9).

### 3.6 Publication bias

**Figure 10:**
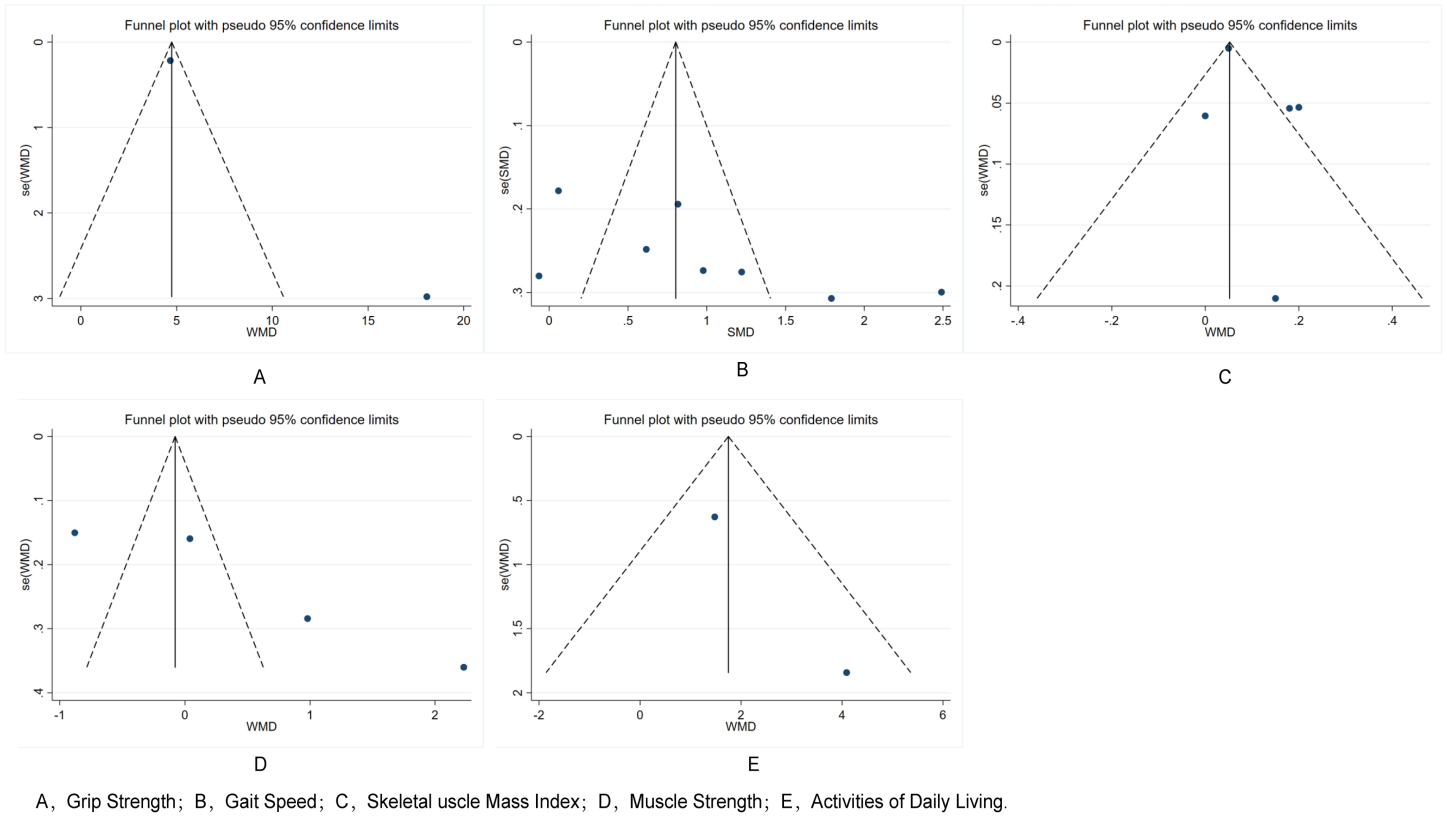
Funnel plots of outcome indicators.

In this study, we conducted a comprehensive assessment of publication bias to ensure the robustness of our conclusions. Funnel plots were generated for the primary outcomes—grip strength, gait speed, skeletal muscle mass index, muscle strength, and activities of daily living—and demonstrated generally symmetrical distributions, suggesting a low likelihood of publication bias (Figure 10). Further, Egger’s test results indicated no significant bias for grip strength (*t* = 0.41, *p* = 0.695), gait speed (*t* = 1.37, *p* = 0.265), or skeletal muscle mass index (*t* = 3.16, *p* = 0.087). Although the *p*-value for skeletal muscle mass index approached 0.05, it remains above the conventional threshold, supporting the conclusion that publication bias has minimal impact on our overall findings.

## 4 Discussion

In aging populations, sarcopenia often leads to a progressive decline in physical function, increasing the risk of falls and loss of independence (8). Tai Chi and Baduanjin, as low-risk, low-cost, non-pharmacological interventions, involve low-intensity, progressive movements that align with the safety requirements of older adults. These modalities reduce the risk of exercise-induced injuries commonly associated with traditional resistance training, making them particularly suitable for community-based and home-based programs.

In this systematic review and meta-analysis of 738 patients with sarcopenia, we found that regular practice of Tai Chi and Baduanjin significantly improved grip strength (SMD = 0.97, 95% CI: 0.42 to 1.52, *Z* = 3.45, *p* = 0.001), gait speed (WMD = 0.10, 95% CI: 0.02 to 0.19, *Z* = 2.47, *p* = 0.013), and muscle strength (WMD = 1.75, 95% CI: 0.59 to 2.91, *Z* = 2.95, *p* = 0.003). Although improvements were also observed in skeletal muscle mass index (WMD = 0.55, 95% CI: −0.54 to 1.65, *Z* = 0.99, *p* = 0.323) and activities of daily living (WMD = 11.04, 95% CI: −2.08 to 24.16, *Z* = 1.65, *p* = 0.099), these changes did not result in a noticeable improvement. Our findings align with a growing body of research supporting the functional benefits of traditional mind–body practices. These exercises not only improve balance and gait but also integrate low-impact movements that promote neuromuscular coordination and proprioceptive awareness (9–14). These exercises are thought to delay the progression of sarcopenia by modulating muscle protein synthesis and degradation, improving nutritional status, enhancing blood circulation, and reducing systemic inflammation (15). Additionally, evidence suggests that traditional practices such as Tai Chi and Baduanjin can activate the PGC-1α/FNDC5/UCP1 signaling pathway, leading to upregulation of PGC-1α, increased mitochondrial biogenesis, muscle fiber hypertrophy, and attenuation of skeletal muscle atrophy. These adaptations likely contribute to enhanced muscle strength and physical function (16). Aging is strongly associated with a decline in skeletal muscle mass and function, which impairs ADL in older adults (17).

In our study, although Tai Chi and Baduanjin showed some improvement in skeletal muscle mass index and ADL compared to control interventions, these effects did not reach a noticeable level of improvement. These findings imply that while mind–body exercises support functional performance, their capacity to induce structural muscle changes may be limited, especially without longer or more intensive protocols. Our findings align with prior studies reporting that Baduanjin failed to produce statistically significant gains in muscle strength among frail older adults (18). Similarly, other low-intensity interventions, such as Tai Chi, have shown no measurable improvements in physical function in community-dwelling older adult populations (19, 20). Several factors may explain these outcomes. First, substantial gains in skeletal muscle mass typically require high-intensity resistance training or prolonged intervention periods. Resistance exercise consistently yields significant improvements in muscle mass, strength, and physical performance (21, 22). In contrast, most studies included in this review implemented moderate-duration interventions (12–24 weeks), and the low mechanical loading of Tai Chi and Baduanjin likely provided insufficient stimulus for muscle hypertrophy. Additionally, some studies employed simplified forms of Tai Chi, which may have resulted in training volumes below the “minimum effective dose” needed to induce muscular adaptation (23, 24). Long-term adherence can be challenging in older adults with sarcopenia, particularly due to mobility issues, comorbidities, and fluctuations in health status (25, 26). Moreover, the optimal training intensity and duration for Baduanjin remain undefined (27), further complicating standardization of these interventions. Beyond structural changes, the benefits of traditional exercise may stem from neuromuscular adaptations. Rather than directly enlarging muscle fiber cross-sectional area, Tai Chi and Baduanjin may enhance neuromuscular coordination, motor unit recruitment, and balance control. These adaptations can lead to functional improvements that precede measurable changes in muscle morphology. For instance, one study demonstrated that 12 weeks of Tai Chi significantly improved gait stability in patients with Parkinson’s disease.

Electromyography (EMG) data demonstrated enhanced muscle coactivation patterns - reflecting improved neuromuscular coordination - without corresponding increases in muscle mass (28). To better capture dynamic changes in muscle morphology, future studies should employ advanced imaging modalities such as magnetic resonance imaging (MRI) or dual-energy X-ray absorptiometry (DXA), which allow for longitudinal monitoring and identification of critical time windows for structural adaptation (29). The absence of significant gains in ADL may reflect its complexity, as daily functioning is influenced not only by physical strength but also by psychological, social, and environmental factors. A single exercise intervention may be insufficient to overcome these confounding influences. Prior studies have shown that in sarcopenia research, variables such as social engagement, chronic disease burden, demographic characteristics, and lifestyle behaviors must be carefully accounted for, as they are strongly associated with both psychological health and physical performance (30). Importantly, our findings highlight the need for a pragmatic approach in resource-limited primary care settings. In such contexts, prioritizing functional metrics - such as gait speed - may be more clinically relevant than focusing exclusively on muscle mass. Functional outcomes are more directly associated with fall prevention, mobility independence, and overall quality of life in patients with sarcopenia. In addition to focusing on pragmatic outcomes, it is equally important to consider how training structure and contextual factors influence intervention effectiveness and adherence. Recent systematic reviews of various Tai Chi interventions indicate that a structured 12-week program practiced 2 to 5 times per week for 60 min per session significantly improves balance, gait stability, physical activity, muscle strength, and fall prevention in older adults. Building on this, multiple studies have highlighted the influence of psychosocial and environmental factors on older adults’ adherence and long-term participation. For instance, perceived social support, self-efficacy, and motivational structures have been shown to strongly impact adherence. Group-based Tai Chi was found to significantly enhance perceived support, contributing to higher participation rates. Similarly, home-based “Tai Chi snacking” interventions improved both physical function and self-efficacy, with participants valuing their flexibility and convenience. Environmental facilitators—such as access to safe training spaces and digital literacy—also play a critical role in program adoption, especially in home-based or remote settings. Thus, future studies should include standardized measures of psychosocial and environmental influences to better capture the full spectrum of factors shaping intervention outcomes.

Tai Chi and Baduanjin, as traditional mind–body exercises rooted in Chinese culture, have shown increasing feasibility and acceptability among older adults in Western settings, providing a realistic foundation for their global dissemination. Firstly, the practice of Tai Chi has been widely accepted in Western communities, with studies demonstrating favorable adherence and safety profiles. For instance, a 12-week Tai Chi intervention conducted in assisted living facilities in the United States reported improvements in fear of falling and functional capacity, without any serious adverse events, and was positively received by participants (31). Additionally, during the COVID-19 pandemic, a remote intervention based on the concept of “Tai Chi snacking”—involving short, dispersed sessions—proved feasible and well-accepted among homebound older adults, while also enhancing their physical function (32). Secondly, although Baduanjin is less well-known outside China, emerging evidence suggests it is both feasible and well-tolerated in non-Chinese populations. A 2020 single-arm feasibility study in Singapore implemented a 16-week Baduanjin program (44 sessions) in frail and pre-frail older adults, achieving an attendance rate of 89% with no training-related adverse events. Participants also reported improvements in grip strength, gait performance, and cognitive function (33). Similarly, a pilot randomized controlled trial among older cancer survivors indicated that Baduanjin, as a low-intensity exercise modality, has promising potential for implementation and high participant acceptability (34). Collectively, these findings indicate that both Tai Chi and Baduanjin can be culturally adapted to enhance engagement in non-Chinese populations, through strategies such as form simplification, online instruction, and emphasis on their low-impact, stress-reducing characteristics. Moreover, previous research has highlighted that culturally tailored exercise programs significantly improve adherence and participation rates (35). To facilitate broader adoption in multicultural settings, future interventions should prioritize the use of standardized and simplified routines, emphasize culturally sensitive instruction and explanation, and integrate community-based with remote delivery platforms to support flexible and sustainable implementation.

This study has several limitations. After assessing the risk of bias in the 11 included studies, only three were classified as low risk. Nevertheless, these studies still offer valuable insights, particularly in areas where sample sizes are limited. To mitigate potential bias, we conducted a sensitivity analysis excluding the high-risk studies, and found that our main conclusions remained robust. This suggests that our results are reasonably stable, though they should still be interpreted with caution. We recommend that future research prioritize methodological rigor and standardized implementation to further reduce bias and enhance the credibility of findings. First, although our meta-analysis included randomized controlled trials (RCTs), the total number of eligible studies per outcome was relatively small. This limitation may reduce the robustness of pooled estimates. Risk of bias assessments revealed concerns in some studies regarding random sequence generation, which may reduce statistical power and increase the potential for biased outcomes (36, 37). Second, while funnel plots appeared symmetrical, the small number of studies included for most outcomes limits the reliability of visual assessments for publication bias. Consequently, the possibility of undetected reporting bias cannot be fully excluded. Third, variations in how the interventions were delivered, such as differences in duration, frequency, and intensity, likely contributed to the inconsistent results seen across studies and may have influenced the reliability of the pooled effect estimates (38). For example, the degree of standardization in Tai Chi and Baduanjin protocols varied across the included studies. While some trials clearly employed validated routines—such as the 24-form Yang-style Tai Chi or the standardized Baduanjin sequences issued by the General Administration of Sport of China, while others used simplified movements to accommodate participants with limited mobility. Variability in intervention content and exercise modalities may have contributed to the heterogeneity observed in the results, particularly in outcomes like skeletal muscle mass and activities of daily living, which are sensitive to both exercise intensity and duration. We also found that heterogeneity in our results may be influenced by baseline participant characteristics. A recent meta-analysis demonstrated that studies adjusting for baseline BMI, age, and diagnostic criteria reported significantly different effect sizes for sarcopenia outcomes (39). Moreover, the follow-up durations in the included studies were relatively short, typically ranging from 8 to 24 weeks. Such timeframes may be insufficient to capture the long-term effects of mind–body interventions on muscle mass, functional capacity, and activities of daily living, especially given that physiological adaptations often require sustained intervention. Future studies should include longer follow-up periods to determine whether the observed benefits are durable over time. In summary, future studies should carefully control both intervention protocols and participants’ baseline characteristics to reduce heterogeneity. Additionally, most studies did not account for common comorbidities in sarcopenic populations—such as osteoporosis and diabetes - in their interaction analyses, which may limit the generalizability of the findings to more clinically complex settings. Sex-specific differences in physiological responses to exercise are well documented (40), yet many studies failed to ensure balanced gender representation. Future trials should strive for gender parity to explore potential sex-based mechanisms underlying the effects of physical activity in sarcopenia. Moreover, our analysis focused only on the short-term effects of Tai Chi and Baduanjin on muscle mass, muscle function, and activities of daily living. Although low-intensity exercise is associated with improved long-term adherence (41), which may lead to sustained health benefits, the long-term effects of these interventions remain unclear. Future research should prioritize large-scale, multicenter RCTs using standardized protocols—such as 24-form simplified Tai Chi or validated Baduanjin routines—and extend the intervention duration to better assess the sustainability of improvements.

## 5 Conclusion

This systematic review and meta-analysis demonstrate that Tai Chi and Baduanjin, as low-intensity mind–body exercise interventions, significantly improve muscle function—specifically grip strength, gait speed, and overall muscle strength - in patients with sarcopenia. However, their effects on skeletal muscle mass index and activities of daily living did not achieve statistical significance. Our findings suggest that traditional exercises likely enhance function through neuromuscular pathways rather than by increasing muscle mass alone. In contrast, inducing measurable structural changes in muscle mass may require longer intervention durations or integrated approaches that combine exercise with nutritional support. Given their safety, accessibility, and functional benefits, Tai Chi and Baduanjin offer promising, low-barrier interventions for older adults with sarcopenia - particularly in primary care and community health settings where high-intensity resistance training may not be feasible. Future trials should apply stricter inclusion criteria and extend follow-up periods to ensure patient baseline homogeneity and capture long-term changes. Objective assessments using imaging modalities (DXA, CT, MRI) and biomarkers should be incorporated to accurately measure muscle composition, architecture, and quality. Moreover, conducting large-scale, multicenter, and cross-cultural studies will be essential to validate the generalizability of these findings across diverse populations.

## Data Availability

The original contributions presented in the study are included in the article/Supplementary material, further inquiries can be directed to the corresponding authors.
